# Tris(5,6-dimethyl-1*H*-benzimidazole-κ*N* ^3^)(pyridine-2,6-dicarboxyl­ato-κ^3^ *O* ^2^,*N*,*O* ^6^)nickel(II)

**DOI:** 10.1107/S1600536812019502

**Published:** 2012-05-05

**Authors:** Yue-Hua Li, Feng-Feng Li, Xin-Hua Liu, Ling-Yan Zhao

**Affiliations:** a College of Chemical Engineering, Hebei United University, Tangshan 063009, People’s Republic of China; b College of Light Industry, Hebei United University, Tangshan 063009, People’s Republic of China; c Qian’an College, Hebei United University, Tangshan 063009, People’s Republic of China

## Abstract

The title mononuclear complex, [Ni(C_7_H_3_NO_4_)(C_9_H_10_N_2_)_3_], shows a central Ni^II^ atom which is coordinated by two carboxyl­ate O atoms and the N atom from a pyridine-2,6-dicarboxyl­ate ligand and by three N atoms from different 5,6-dimethyl-1*H*-­benzimidazole ligands in a distorted octa­hedral geometry. The crystal structure shows intermolecular N—H⋯O hydrogen bonds.

## Related literature

For related structures of dipicolinate complexes, see: How *et al.* (1991 ▶); Dong *et al.* (2010 ▶); Liu *et al.* (2011 ▶).
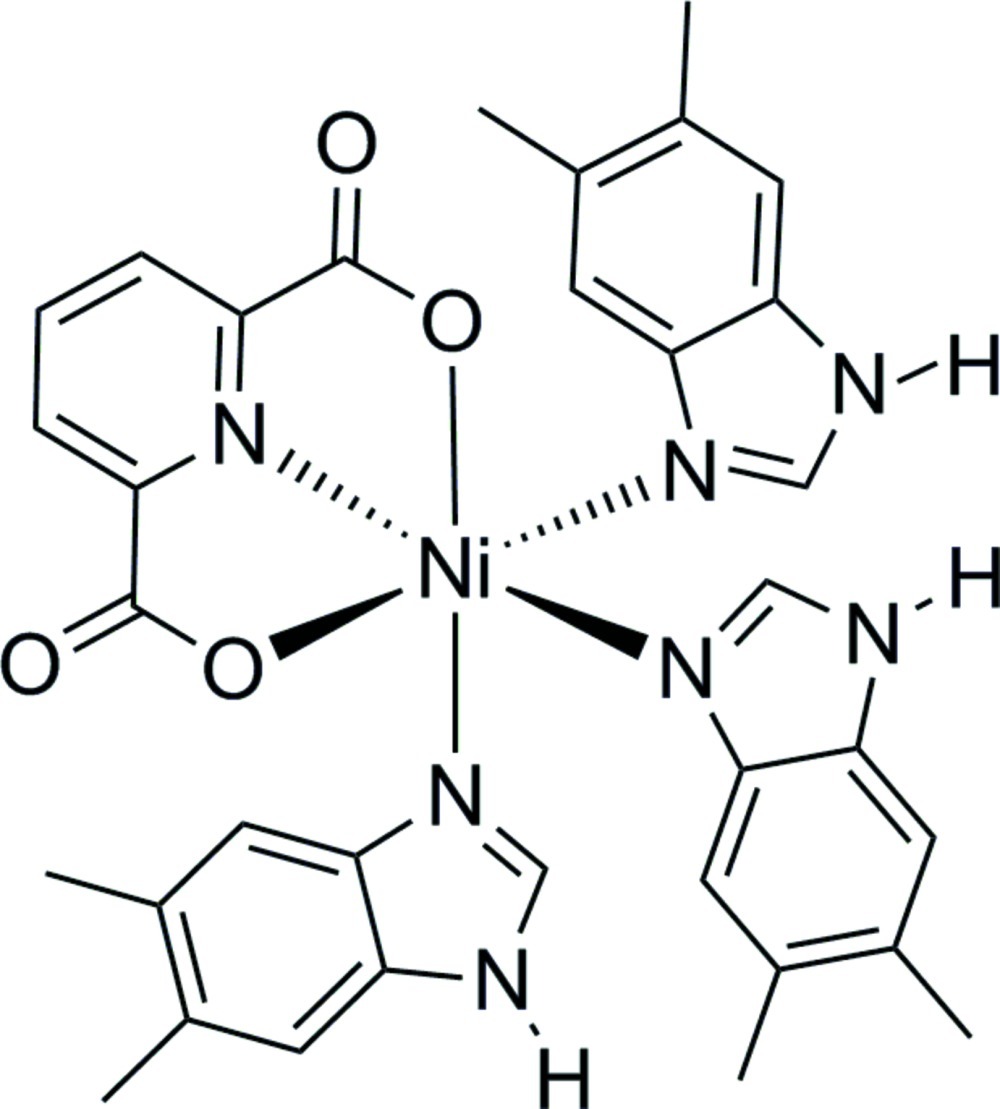


## Experimental

### 

#### Crystal data

[Ni(C_7_H_3_NO_4_)(C_9_H_10_N_2_)_3_]
*M* *_r_* = 662.38
Triclinic, 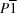

*a* = 7.5884 (7) Å
*b* = 9.4499 (9) Å
*c* = 23.839 (2) Å
α = 89.0040 (9)°
β = 81.484 (1)°
γ = 74.078 (1)°
*V* = 1625.3 (3) Å^3^
*Z* = 2
Mo *K*α radiation
μ = 0.65 mm^−1^
*T* = 293 K
0.18 × 0.15 × 0.14 mm

#### Data collection

Bruker APEXII CCD diffractometer
Absorption correction: multi-scan (*SADABS*; Sheldrick, 1996 ▶) *T*_min_ = 0.872, *T*_max_ = 0.935
12396 measured reflections
5721 independent reflections
5078 reflections with *I* > 2σ(*I*)
*R*_int_ = 0.021

#### Refinement

*R*[*F* ^2^ > 2σ(*F* ^2^)] = 0.032
*wR*(*F* ^2^) = 0.087
*S* = 0.94
5721 reflections
421 parameters
H-atom parameters constrained
Δρ_max_ = 0.27 e Å^−3^
Δρ_min_ = −0.34 e Å^−3^

### 

Data collection: *APEX2* (Bruker, 2007 ▶); cell refinement: *SAINT* (Bruker, 2007 ▶); data reduction: *SAINT*; program(s) used to solve structure: *SHELXS97* (Sheldrick, 2008 ▶); program(s) used to refine structure: *SHELXL97* (Sheldrick, 2008 ▶); molecular graphics: *SHELXTL* (Sheldrick, 2008 ▶); software used to prepare material for publication: *SHELXTL*.

## Supplementary Material

Crystal structure: contains datablock(s) I, global. DOI: 10.1107/S1600536812019502/im2371sup1.cif

Structure factors: contains datablock(s) I. DOI: 10.1107/S1600536812019502/im2371Isup2.hkl

Additional supplementary materials:  crystallographic information; 3D view; checkCIF report

## Figures and Tables

**Table 1:** Hydrogen-bond geometry (Å, °)

*D*—H⋯*A*	*D*—H	H⋯*A*	*D*⋯*A*	*D*—H⋯*A*
N3—H3*A*⋯O4^i^	0.86	2.27	2.877 (2)	127
N5—H5*A*⋯O1^ii^	0.86	2.14	2.786 (2)	132
N7—H7*A*⋯O4^iii^	0.86	1.94	2.788 (2)	171

Symmetry codes: (i) 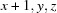; (ii) 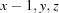; (iii) 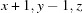.

**Fig. 1.**
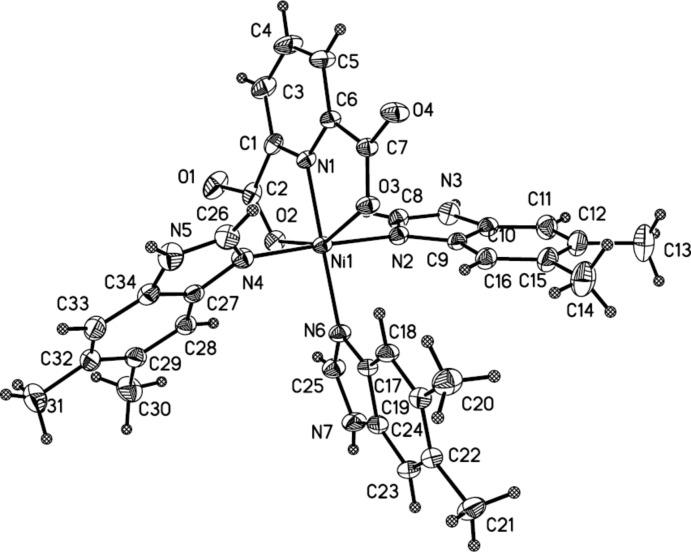
Molecular structure of the title compound with displacement ellipsoids drawn at the 30% probability level. H atoms are presented as a small spheres of arbitrary radius.

## supplementary crystallographic information

### Comment

Benzimidazole and its derivatives are widely used as intermediates in synthesis and commonly coordinate to transition metals by N atom. Some Cu(II) dipicolinate complexes with additional imidazole ligands have been reported (How *et al.*, 1991; Dong *et al.*, 2010; Liu *et al.*, 2011). Here, we report the crystal structure of the title compound.

The asymmetric unit of (I) contains one nickel(II), one 2,6- pyridinedicarboxylato and three 5,6-dimethylbenzimidazole ligands. The nickel center is six-coordinated in a distorted octahedral coordination geometry (Fig.1). Each nickel(II) is coordinated by one tridentate dipicolinato ligand *via* its carboxylate oxygen atoms and the pyridine nitrogen atom as donors (Ni1—N1= 1.997 (2); Ni1—O2=2.134 (1); Ni1—O3= 2.190 (1) Å). Three additional N donor atoms from three 5,6-dimethylbenzimidazole ligands complete the coordination sphere of nickel (Ni1- N from 2.056 (2) to 2.123 (2) Å). It is noteworthy that there exist strong N—H···O hydrogen bond interactions (Table 1) involving the carboxy group oxygen atoms of dipicolinato ligands as well as the NH functions of the benzimidazole ligands.

### Experimental

A mixture of Ni(NO_3_)_2_.6H_2_O (290.8 mg, 0.5 mmol), 2,6-pyridinedicarboxylic acid (167 mg, 1 mmol), NaOH (80 mg, 2 mm mol), 5,6-dimethylbenzimidazole (73 mg, 0.5 mmol) and water (12 ml) was sealed in a 25 ml teflon-lined stainless steel reactor and heated to 413 K for 72 h. The reaction was cooled to room temperature over a period of 24 h. Green prismatic crystals of (I) suitable for *X*– ray difraction analysis were obtained with a yield of 38% (based Ni(NO_3_)_2_.6H_2_O).

### Refinement

All H atoms were positioned geometrically and refined using a riding model with C—H = 0.93 Å and *U*_iso_(H) = 1.2*U*_eq_(C) for aromatic H, N—H = 0.86 Å and *U*_iso_(H) = 1.2*U*_eq_(N) for the NH group.

### Crystal data

**Table:**

[Ni(C_7_H_3_NO_4_)(C_9_H_10_N_2_)_3_]	*V* = 1625.3 (3) Å^3^
*M**_r_* = 662.38	*Z* = 2
Triclinic, *P*1	*F*(000) = 692
Hall symbol: -P 1	*D*_x_ = 1.353 Mg m^−^^3^
*a* = 7.5884 (7) Å	Mo *K*α radiation, λ = 0.71073 Å
*b* = 9.4499 (9) Å	µ = 0.65 mm^−^^1^
*c* = 23.839 (2) Å	*T* = 293 K
α = 89.0040 (9)°	Block, green
β = 81.484 (1)°	0.18 × 0.15 × 0.14 mm
γ = 74.078 (1)°	

### Data collection

**Table:**

Bruker APEXII CCD diffractometer	5721 independent reflections
Radiation source: fine-focus sealed tube	5078 reflections with *I* > 2σ(*I*)
Graphite monochromator	*R*_int_ = 0.021
φ and ω scans	θ_max_ = 25.0°, θ_min_ = 2.2°
Absorption correction: multi-scan (*SADABS*; Sheldrick, 1996)	*h* = −9→9
*T*_min_ = 0.872, *T*_max_ = 0.935	*k* = −11→11
12396 measured reflections	*l* = −28→28

### Refinement

**Table:**

Refinement on *F*^2^	Primary atom site location: structure-invariant direct methods
Least-squares matrix: full	Secondary atom site location: difference Fourier map
*R*[*F*^2^ > 2σ(*F*^2^)] = 0.032	Hydrogen site location: inferred from neighbouring sites
*wR*(*F*^2^) = 0.087	H-atom parameters constrained
*S* = 0.94	*w* = 1/[σ^2^(*F*_o_^2^) + (0.0503*P*)^2^ + 0.7157*P*] where *P* = (*F*_o_^2^ + 2*F*_c_^2^)/3
5721 reflections	(Δ/σ)_max_ = 0.001
421 parameters	Δρ_max_ = 0.27 e Å^−^^3^
0 restraints	Δρ_min_ = −0.34 e Å^−^^3^

### Special details

**Table:**

Geometry. All e.s.d.'s (except the e.s.d. in the dihedral angle between two l.s. planes) are estimated using the full covariance matrix. The cell e.s.d.'s are taken into account individually in the estimation of e.s.d.'s in distances, angles and torsion angles; correlations between e.s.d.'s in cell parameters are only used when they are defined by crystal symmetry. An approximate (isotropic) treatment of cell e.s.d.'s is used for estimating e.s.d.'s involving l.s. planes.
Refinement. Refinement of *F*^2^ against ALL reflections. The weighted *R*-factor *wR* and goodness of fit *S* are based on *F*^2^, conventional *R*-factors *R* are based on *F*, with *F* set to zero for negative *F*^2^. The threshold expression of *F*^2^ > σ(*F*^2^) is used only for calculating *R*-factors(gt) *etc*. and is not relevant to the choice of reflections for refinement. *R*-factors based on *F*^2^ are statistically about twice as large as those based on *F*, and *R*- factors based on ALL data will be even larger.

### Fractional atomic coordinates and isotropic or equivalent isotropic displacement parameters (Å^2^)

**Table:**

	*x*	*y*	*z*	*U*_iso_*/*U*_eq_	
Ni1	0.99661 (3)	0.63280 (2)	0.222282 (9)	0.02907 (9)	
N1	0.9467 (2)	0.84068 (16)	0.19677 (6)	0.0297 (3)	
N4	0.8878 (2)	0.57135 (18)	0.15400 (7)	0.0365 (4)	
N2	1.1152 (2)	0.68619 (17)	0.29151 (7)	0.0342 (4)	
N6	1.0510 (2)	0.41948 (17)	0.24934 (7)	0.0362 (4)	
O3	0.71968 (17)	0.73993 (13)	0.26733 (6)	0.0349 (3)	
O2	1.24640 (17)	0.62840 (15)	0.16685 (6)	0.0389 (3)	
O4	0.51849 (18)	0.96040 (15)	0.28495 (7)	0.0459 (4)	
C23	0.9603 (3)	0.1205 (2)	0.33262 (9)	0.0443 (5)	
H23	1.0312	0.0285	0.3419	0.053*	
C7	0.6658 (2)	0.87699 (19)	0.26080 (8)	0.0313 (4)	
C6	0.7936 (2)	0.9412 (2)	0.21938 (8)	0.0318 (4)	
C25	1.2158 (3)	0.3253 (2)	0.24373 (9)	0.0423 (5)	
H25	1.3208	0.3441	0.2232	0.051*	
C9	1.0555 (3)	0.6914 (2)	0.35000 (8)	0.0334 (4)	
C1	1.0729 (3)	0.8747 (2)	0.15801 (8)	0.0351 (4)	
O1	1.3415 (2)	0.7556 (2)	0.09431 (7)	0.0637 (5)	
N7	1.2178 (2)	0.19856 (18)	0.27062 (8)	0.0453 (4)	
H7A	1.3124	0.1237	0.2712	0.054*	
C17	0.9343 (3)	0.3497 (2)	0.28265 (8)	0.0337 (4)	
N3	1.3074 (2)	0.7679 (2)	0.33549 (7)	0.0439 (4)	
H3A	1.4004	0.7997	0.3400	0.053*	
C8	1.2639 (3)	0.7329 (2)	0.28601 (9)	0.0392 (5)	
H8	1.3330	0.7411	0.2511	0.047*	
C2	1.2345 (3)	0.7425 (2)	0.13747 (8)	0.0393 (5)	
C27	0.9756 (3)	0.4677 (2)	0.11018 (8)	0.0343 (4)	
C18	0.7458 (3)	0.3971 (2)	0.30265 (8)	0.0370 (4)	
H18	0.6745	0.4876	0.2922	0.044*	
C28	1.1593 (3)	0.3843 (2)	0.09756 (8)	0.0389 (4)	
H28	1.2472	0.3954	0.1192	0.047*	
C24	1.0398 (3)	0.2113 (2)	0.29699 (8)	0.0385 (5)	
C16	0.9040 (3)	0.6554 (2)	0.38086 (9)	0.0408 (5)	
H16	0.8247	0.6195	0.3625	0.049*	
C19	0.6656 (3)	0.3076 (2)	0.33837 (9)	0.0392 (5)	
C34	0.8467 (3)	0.4538 (2)	0.07628 (9)	0.0400 (5)	
C10	1.1749 (3)	0.7435 (2)	0.37780 (9)	0.0389 (4)	
N5	0.6813 (2)	0.5511 (2)	0.10001 (8)	0.0508 (5)	
H5A	0.5765	0.5671	0.0879	0.061*	
C22	0.7744 (3)	0.1691 (2)	0.35405 (9)	0.0426 (5)	
C29	1.2092 (3)	0.2845 (2)	0.05225 (9)	0.0441 (5)	
C5	0.7630 (3)	1.0872 (2)	0.20455 (10)	0.0462 (5)	
H5	0.6570	1.1581	0.2207	0.055*	
C3	1.0500 (3)	1.0176 (3)	0.14083 (10)	0.0513 (6)	
H3	1.1374	1.0417	0.1135	0.062*	
C26	0.7146 (3)	0.6156 (2)	0.14517 (9)	0.0457 (5)	
H26	0.6236	0.6855	0.1682	0.055*	
C14	0.7053 (4)	0.6362 (4)	0.47250 (12)	0.0769 (8)	
H14A	0.6200	0.7242	0.4900	0.115*	
H14B	0.7447	0.5672	0.5013	0.115*	
H14C	0.6455	0.5934	0.4473	0.115*	
C32	1.0753 (4)	0.2693 (2)	0.01906 (9)	0.0498 (6)	
C33	0.8945 (3)	0.3547 (3)	0.03046 (9)	0.0502 (5)	
H33	0.8070	0.3464	0.0082	0.060*	
C15	0.8732 (3)	0.6738 (3)	0.43916 (9)	0.0496 (5)	
C11	1.1441 (3)	0.7635 (3)	0.43657 (9)	0.0519 (6)	
H11	1.2237	0.7997	0.4548	0.062*	
C31	1.1298 (5)	0.1571 (3)	−0.02934 (11)	0.0750 (8)	
H31A	1.0250	0.1635	−0.0484	0.112*	
H31B	1.1704	0.0601	−0.0147	0.112*	
H31C	1.2287	0.1765	−0.0556	0.112*	
C12	0.9932 (4)	0.7287 (3)	0.46718 (9)	0.0554 (6)	
C4	0.8950 (4)	1.1245 (3)	0.16495 (11)	0.0579 (6)	
H4	0.8790	1.2220	0.1546	0.069*	
C20	0.4618 (3)	0.3609 (3)	0.36204 (11)	0.0574 (6)	
H20A	0.4480	0.3879	0.4014	0.086*	
H20B	0.4040	0.2837	0.3582	0.086*	
H20C	0.4039	0.4449	0.3415	0.086*	
C13	0.9562 (5)	0.7524 (4)	0.53117 (11)	0.0908 (10)	
H13A	1.0543	0.7846	0.5433	0.136*	
H13B	0.9503	0.6616	0.5491	0.136*	
H13C	0.8405	0.8258	0.5416	0.136*	
C21	0.6875 (4)	0.0748 (3)	0.39503 (11)	0.0608 (7)	
H21A	0.7793	−0.0147	0.4009	0.091*	
H21B	0.5884	0.0518	0.3796	0.091*	
H21C	0.6395	0.1274	0.4306	0.091*	
C30	1.4084 (4)	0.1915 (3)	0.03947 (11)	0.0643 (7)	
H30A	1.4795	0.2179	0.0656	0.096*	
H30B	1.4592	0.2084	0.0014	0.096*	
H30C	1.4128	0.0894	0.0432	0.096*	

### Atomic displacement parameters (Å^2^)

**Table:**

	*U*^11^	*U*^22^	*U*^33^	*U*^12^	*U*^13^	*U*^23^
Ni1	0.02265 (13)	0.02596 (14)	0.03356 (14)	−0.00152 (9)	0.00148 (9)	0.00487 (9)
N1	0.0260 (8)	0.0300 (8)	0.0320 (8)	−0.0065 (6)	−0.0031 (6)	0.0069 (6)
N4	0.0296 (8)	0.0356 (9)	0.0405 (9)	−0.0043 (7)	−0.0027 (7)	0.0024 (7)
N2	0.0307 (8)	0.0341 (8)	0.0354 (9)	−0.0071 (7)	−0.0015 (7)	0.0052 (7)
N6	0.0329 (9)	0.0284 (8)	0.0421 (9)	−0.0010 (7)	−0.0037 (7)	0.0052 (7)
O3	0.0266 (6)	0.0282 (7)	0.0438 (8)	−0.0027 (5)	0.0035 (5)	0.0076 (6)
O2	0.0274 (7)	0.0441 (8)	0.0395 (8)	−0.0039 (6)	0.0022 (6)	0.0033 (6)
O4	0.0290 (7)	0.0337 (8)	0.0642 (10)	0.0022 (6)	0.0063 (7)	0.0032 (7)
C23	0.0577 (14)	0.0256 (10)	0.0444 (12)	−0.0020 (9)	−0.0101 (10)	0.0061 (9)
C7	0.0239 (9)	0.0286 (10)	0.0383 (10)	−0.0027 (7)	−0.0034 (8)	0.0021 (8)
C6	0.0295 (9)	0.0275 (9)	0.0364 (10)	−0.0046 (7)	−0.0052 (8)	0.0052 (8)
C25	0.0344 (11)	0.0362 (11)	0.0489 (12)	−0.0003 (9)	−0.0014 (9)	0.0053 (9)
C9	0.0337 (10)	0.0284 (9)	0.0345 (10)	−0.0037 (8)	−0.0036 (8)	0.0041 (8)
C1	0.0314 (10)	0.0448 (11)	0.0312 (10)	−0.0152 (8)	−0.0038 (8)	0.0096 (8)
O1	0.0379 (8)	0.0897 (13)	0.0485 (9)	−0.0043 (8)	0.0132 (7)	0.0230 (9)
N7	0.0388 (10)	0.0315 (9)	0.0539 (11)	0.0081 (7)	−0.0040 (8)	0.0064 (8)
C17	0.0397 (10)	0.0241 (9)	0.0354 (10)	−0.0049 (8)	−0.0071 (8)	0.0027 (8)
N3	0.0341 (9)	0.0525 (11)	0.0479 (10)	−0.0169 (8)	−0.0058 (8)	0.0023 (8)
C8	0.0336 (10)	0.0428 (11)	0.0391 (11)	−0.0106 (9)	0.0006 (8)	0.0053 (9)
C2	0.0257 (10)	0.0573 (13)	0.0335 (10)	−0.0108 (9)	−0.0011 (8)	0.0072 (9)
C27	0.0384 (10)	0.0300 (10)	0.0337 (10)	−0.0101 (8)	−0.0026 (8)	0.0073 (8)
C18	0.0364 (10)	0.0260 (9)	0.0441 (11)	−0.0006 (8)	−0.0071 (9)	0.0038 (8)
C28	0.0390 (11)	0.0367 (11)	0.0387 (11)	−0.0091 (9)	−0.0010 (9)	0.0034 (8)
C24	0.0424 (11)	0.0283 (10)	0.0394 (11)	0.0003 (8)	−0.0082 (9)	0.0006 (8)
C16	0.0403 (11)	0.0423 (11)	0.0398 (11)	−0.0138 (9)	−0.0016 (9)	0.0052 (9)
C19	0.0439 (11)	0.0338 (10)	0.0394 (11)	−0.0105 (9)	−0.0047 (9)	0.0006 (8)
C34	0.0427 (11)	0.0402 (11)	0.0395 (11)	−0.0150 (9)	−0.0081 (9)	0.0096 (9)
C10	0.0370 (11)	0.0362 (11)	0.0415 (11)	−0.0070 (8)	−0.0059 (9)	0.0045 (9)
N5	0.0359 (10)	0.0623 (12)	0.0540 (11)	−0.0091 (9)	−0.0143 (8)	0.0018 (9)
C22	0.0581 (14)	0.0325 (11)	0.0369 (11)	−0.0130 (10)	−0.0055 (10)	0.0033 (9)
C29	0.0530 (13)	0.0370 (11)	0.0373 (11)	−0.0109 (10)	0.0059 (9)	0.0028 (9)
C5	0.0465 (12)	0.0288 (10)	0.0581 (14)	−0.0048 (9)	−0.0032 (10)	0.0081 (9)
C3	0.0533 (14)	0.0510 (13)	0.0524 (13)	−0.0238 (11)	−0.0008 (11)	0.0204 (11)
C26	0.0324 (11)	0.0499 (13)	0.0494 (13)	−0.0031 (9)	−0.0041 (9)	−0.0001 (10)
C14	0.0722 (19)	0.104 (2)	0.0523 (15)	−0.0346 (17)	0.0154 (13)	0.0067 (15)
C32	0.0753 (16)	0.0429 (12)	0.0329 (11)	−0.0229 (12)	−0.0008 (11)	0.0032 (9)
C33	0.0646 (15)	0.0521 (13)	0.0414 (12)	−0.0255 (12)	−0.0141 (11)	0.0078 (10)
C15	0.0527 (13)	0.0550 (14)	0.0378 (11)	−0.0146 (11)	0.0026 (10)	0.0069 (10)
C11	0.0561 (14)	0.0587 (14)	0.0437 (12)	−0.0166 (11)	−0.0150 (11)	−0.0005 (10)
C31	0.105 (2)	0.0683 (18)	0.0490 (15)	−0.0240 (17)	−0.0016 (15)	−0.0136 (13)
C12	0.0630 (15)	0.0625 (15)	0.0365 (12)	−0.0121 (12)	−0.0042 (11)	0.0030 (11)
C4	0.0665 (16)	0.0356 (12)	0.0719 (16)	−0.0181 (11)	−0.0056 (13)	0.0220 (11)
C20	0.0497 (14)	0.0542 (14)	0.0639 (15)	−0.0128 (11)	0.0020 (11)	0.0094 (12)
C13	0.107 (3)	0.128 (3)	0.0393 (15)	−0.039 (2)	−0.0024 (15)	−0.0056 (16)
C21	0.0778 (18)	0.0452 (14)	0.0577 (15)	−0.0200 (12)	−0.0007 (13)	0.0167 (11)
C30	0.0601 (15)	0.0596 (16)	0.0592 (15)	−0.0043 (12)	0.0143 (12)	−0.0126 (12)

### Geometric parameters (Å, º)

**Table:**

Ni1—N1	1.9976 (15)	C16—H16	0.9300
Ni1—N6	2.0561 (15)	C19—C22	1.420 (3)
Ni1—N4	2.0886 (16)	C19—C20	1.513 (3)
Ni1—N2	2.1227 (16)	C34—C33	1.390 (3)
Ni1—O2	2.1348 (13)	C34—N5	1.384 (3)
Ni1—O3	2.1901 (12)	C10—C11	1.393 (3)
N1—C6	1.330 (2)	N5—C26	1.335 (3)
N1—C1	1.331 (2)	N5—H5A	0.8600
N4—C26	1.313 (3)	C22—C21	1.511 (3)
N4—C27	1.402 (2)	C29—C32	1.414 (3)
N2—C8	1.309 (3)	C29—C30	1.515 (3)
N2—C9	1.398 (2)	C5—C4	1.386 (3)
N6—C25	1.310 (2)	C5—H5	0.9300
N6—C17	1.394 (2)	C3—C4	1.379 (3)
O3—C7	1.261 (2)	C3—H3	0.9300
O2—C2	1.265 (2)	C26—H26	0.9300
O4—C7	1.243 (2)	C14—C15	1.523 (3)
C23—C22	1.379 (3)	C14—H14A	0.9600
C23—C24	1.389 (3)	C14—H14B	0.9600
C23—H23	0.9300	C14—H14C	0.9600
C7—C6	1.517 (3)	C32—C33	1.378 (3)
C6—C5	1.384 (3)	C32—C31	1.512 (3)
C25—N7	1.346 (3)	C33—H33	0.9300
C25—H25	0.9300	C15—C12	1.413 (3)
C9—C10	1.391 (3)	C11—C12	1.379 (3)
C9—C16	1.391 (3)	C11—H11	0.9300
C1—C3	1.377 (3)	C31—H31A	0.9600
C1—C2	1.518 (3)	C31—H31B	0.9600
O1—C2	1.239 (2)	C31—H31C	0.9600
N7—C24	1.377 (3)	C12—C13	1.519 (3)
N7—H7A	0.8600	C4—H4	0.9300
C17—C18	1.389 (3)	C20—H20A	0.9600
C17—C24	1.400 (3)	C20—H20B	0.9600
N3—C8	1.341 (3)	C20—H20C	0.9600
N3—C10	1.379 (3)	C13—H13A	0.9600
N3—H3A	0.8600	C13—H13B	0.9600
C8—H8	0.9300	C13—H13C	0.9600
C27—C28	1.391 (3)	C21—H21A	0.9600
C27—C34	1.391 (3)	C21—H21B	0.9600
C18—C19	1.384 (3)	C21—H21C	0.9600
C18—H18	0.9300	C30—H30A	0.9600
C28—C29	1.385 (3)	C30—H30B	0.9600
C28—H28	0.9300	C30—H30C	0.9600
C16—C15	1.381 (3)		
			
N1—Ni1—N6	179.06 (6)	C22—C19—C20	120.27 (19)
N1—Ni1—N4	91.46 (6)	C33—C34—N5	133.1 (2)
N6—Ni1—N4	89.43 (6)	C33—C34—C27	121.8 (2)
N1—Ni1—N2	90.59 (6)	N5—C34—C27	105.10 (18)
N6—Ni1—N2	88.51 (6)	N3—C10—C9	105.30 (17)
N4—Ni1—N2	177.57 (6)	N3—C10—C11	133.4 (2)
N1—Ni1—O2	77.98 (6)	C9—C10—C11	121.2 (2)
N6—Ni1—O2	101.73 (6)	C26—N5—C34	107.34 (17)
N4—Ni1—O2	89.53 (6)	C26—N5—H5A	126.3
N2—Ni1—O2	89.62 (6)	C34—N5—H5A	126.3
N1—Ni1—O3	76.12 (5)	C23—C22—C19	120.33 (19)
N6—Ni1—O3	104.15 (6)	C23—C22—C21	119.6 (2)
N4—Ni1—O3	92.00 (6)	C19—C22—C21	120.1 (2)
N2—Ni1—O3	89.76 (6)	C28—C29—C32	120.3 (2)
O2—Ni1—O3	154.09 (5)	C28—C29—C30	118.8 (2)
C6—N1—C1	121.90 (16)	C32—C29—C30	120.9 (2)
C6—N1—Ni1	120.19 (12)	C6—C5—C4	118.0 (2)
C1—N1—Ni1	117.88 (13)	C6—C5—H5	121.0
C26—N4—C27	104.39 (17)	C4—C5—H5	121.0
C26—N4—Ni1	126.53 (14)	C1—C3—C4	118.7 (2)
C27—N4—Ni1	128.93 (13)	C1—C3—H3	120.7
C8—N2—C9	104.53 (16)	C4—C3—H3	120.7
C8—N2—Ni1	124.08 (14)	N4—C26—N5	113.77 (19)
C9—N2—Ni1	131.26 (13)	N4—C26—H26	123.1
C25—N6—C17	105.31 (16)	N5—C26—H26	123.1
C25—N6—Ni1	124.83 (14)	C15—C14—H14A	109.5
C17—N6—Ni1	129.39 (12)	C15—C14—H14B	109.5
C7—O3—Ni1	114.55 (11)	H14A—C14—H14B	109.5
C2—O2—Ni1	113.29 (12)	C15—C14—H14C	109.5
C22—C23—C24	118.82 (18)	H14A—C14—H14C	109.5
C22—C23—H23	120.6	H14B—C14—H14C	109.5
C24—C23—H23	120.6	C33—C32—C29	120.9 (2)
O4—C7—O3	125.25 (17)	C33—C32—C31	119.2 (2)
O4—C7—C6	118.94 (16)	C29—C32—C31	119.9 (2)
O3—C7—C6	115.81 (15)	C32—C33—C34	118.1 (2)
N1—C6—C5	120.68 (18)	C32—C33—H33	121.0
N1—C6—C7	112.95 (15)	C34—C33—H33	121.0
C5—C6—C7	126.36 (17)	C16—C15—C12	120.6 (2)
N6—C25—N7	113.26 (19)	C16—C15—C14	118.6 (2)
N6—C25—H25	123.4	C12—C15—C14	120.7 (2)
N7—C25—H25	123.4	C12—C11—C10	118.6 (2)
C10—C9—C16	120.18 (18)	C12—C11—H11	120.7
C10—C9—N2	109.36 (17)	C10—C11—H11	120.7
C16—C9—N2	130.46 (18)	C32—C31—H31A	109.5
N1—C1—C3	120.40 (19)	C32—C31—H31B	109.5
N1—C1—C2	112.89 (16)	H31A—C31—H31B	109.5
C3—C1—C2	126.71 (18)	C32—C31—H31C	109.5
C25—N7—C24	107.06 (16)	H31A—C31—H31C	109.5
C25—N7—H7A	126.5	H31B—C31—H31C	109.5
C24—N7—H7A	126.5	C11—C12—C15	120.3 (2)
C18—C17—N6	131.03 (17)	C11—C12—C13	119.2 (2)
C18—C17—C24	120.27 (18)	C15—C12—C13	120.5 (2)
N6—C17—C24	108.68 (17)	C3—C4—C5	120.3 (2)
C8—N3—C10	107.15 (17)	C3—C4—H4	119.8
C8—N3—H3A	126.4	C5—C4—H4	119.8
C10—N3—H3A	126.4	C19—C20—H20A	109.5
N2—C8—N3	113.66 (18)	C19—C20—H20B	109.5
N2—C8—H8	123.2	H20A—C20—H20B	109.5
N3—C8—H8	123.2	C19—C20—H20C	109.5
O1—C2—O2	126.2 (2)	H20A—C20—H20C	109.5
O1—C2—C1	118.03 (19)	H20B—C20—H20C	109.5
O2—C2—C1	115.77 (16)	C12—C13—H13A	109.5
C28—C27—C34	119.94 (18)	C12—C13—H13B	109.5
C28—C27—N4	130.66 (18)	H13A—C13—H13B	109.5
C34—C27—N4	109.40 (17)	C12—C13—H13C	109.5
C19—C18—C17	119.02 (17)	H13A—C13—H13C	109.5
C19—C18—H18	120.5	H13B—C13—H13C	109.5
C17—C18—H18	120.5	C22—C21—H21A	109.5
C29—C28—C27	119.0 (2)	C22—C21—H21B	109.5
C29—C28—H28	120.5	H21A—C21—H21B	109.5
C27—C28—H28	120.5	C22—C21—H21C	109.5
N7—C24—C23	133.22 (18)	H21A—C21—H21C	109.5
N7—C24—C17	105.66 (17)	H21B—C21—H21C	109.5
C23—C24—C17	121.09 (19)	C29—C30—H30A	109.5
C15—C16—C9	119.0 (2)	C29—C30—H30B	109.5
C15—C16—H16	120.5	H30A—C30—H30B	109.5
C9—C16—H16	120.5	C29—C30—H30C	109.5
C18—C19—C22	120.42 (19)	H30A—C30—H30C	109.5
C18—C19—C20	119.28 (18)	H30B—C30—H30C	109.5

### Hydrogen-bond geometry (Å, º)

**Table:**

*D*—H···*A*	*D*—H	H···*A*	*D*···*A*	*D*—H···*A*
N3—H3*A*···O4^i^	0.86	2.27	2.877 (2)	127
N5—H5*A*···O1^ii^	0.86	2.14	2.786 (2)	132
N7—H7*A*···O4^iii^	0.86	1.94	2.788 (2)	171

Symmetry codes: (i) *x*+1, *y*, *z*; (ii) *x*−1, *y*, *z*; (iii) *x*+1, *y*−1, *z*.
